# Influence of a programme for prevention of early childhood caries on early orthodontic treatment needs

**DOI:** 10.1007/s00784-020-03295-4

**Published:** 2020-05-07

**Authors:** Yvonne Wagner, I. Knaup, T. J. Knaup, C. Jacobs, M. Wolf

**Affiliations:** 1 Department of Orthodontics, Section Preventive Dentistry and Paediatric Dentistry, Jena University Hospital, An der alten Post 4, Jena, Germany; 2 Department of Orthodontics, RWTH Aachen University Hospital, Aachen, Germany

**Keywords:** Early childhood caries, Prevention, Children, Tooth loss, Orthodontic treatment

## Abstract

**Objectives:**

The aim of this prospective birth cohort study was to evaluate the effect of the programme for prevention (PP) of early childhood caries and the resulting need for orthodontic treatment in 8-year-old German children.

**Material and methods:**

Children who had been enrolled in a caries-risk-related recall system with continuous dental care starting at the time of birth (prevention group, PG) were compared with children of the same birth cohort whose parents decided not to participate in the programme (control group, CG). All children (*n* = 289) participating in the last PP evaluation at the age of 5 years were invited again and examined by blinded clinicians. Dental caries was scored using the WHO diagnostic criteria expanded to d1-level without radiography. Impressions were taken of children with premature tooth loss to analyse space conditions.

**Results:**

Two hundred twenty-seven children (mean age 8.4 ± 0.6 years; 46.7% female) were examined. Children in the PG (*n* = 127) showed significantly lower caries prevalence and experience (3.1%, 0.4 ± 1.0 d_3–4_mft) than children in the CG (37.3%, 3.9 ± 3.5 d_3–4_mft). Orthodontic analysis found a higher prevalence of premature tooth extraction, followed by a greater extent of space loss in the CG (41.0%; 3.3 ± 4.4 mm) vs. PG (7.9%; 0.4 ± 1.9 mm) and an increase in early orthodontic treatment need (KIG P3, IOTN 5).

**Conclusions:**

The PP was an effective approach for preventing caries-related premature tooth loss in children and conserving relevant arch length.

**Clinical relevance:**

Children who received continuous dental care starting at the time of birth showed better oral health with less premature loss of deciduous teeth and lower need for orthodontic treatment at the age of 8 years.

**Trial registration:**

German Clinical Trials Register DRKS00003438, https://drks-neu.uniklinik-freiburg.de/drks_web/navigate.do?navigationId=trial.HTML&TRIAL_ID=DRKS00003438

## Introduction

According to the International Association of Paediatric Dentistry Bangkok Declaration 2019, early childhood caries (ECC) is a common, mostly untreated tooth decay in preschool children with profound impact on child’s lives [1]. The complex multifactorial disease is defined by the American Academy of Pediatric Dentistry (AAPD) as the occurrence of one or more decayed (non-cavitated and cavitated lesions), missing or filled tooth surfaces in any primary tooth in a child under the age of six [2]. Age-specific dietary patterns like frequent sugar exposure through sweetened drinks and foods and bottle or breastfeeding beyond 12 months are the main risk factors for development of ECC [1–4]. Children with ECC may suffer from dental pain, tooth destruction and odontogenic infections [1–4]. Premature loss of deciduous teeth, lack of space and a higher risk of caries development in the permanent dentition are considered to be possible long-term consequences of ECC, even though the available data is limited [1–17]. Primary maxillary incisors and the first and second molars are the teeth often affected by ECC-caused tooth loss [2–4, 8–17]. Premature loss of primary teeth may affect alignment of permanent teeth [8–10]. While the premature loss of anterior teeth causes no space loss, the early loss of primary molars may result in reduced arch length and is worst when tooth loss occurs before eruption of the permanent first molar [8–16]. Tooth migration, crowding, rotations, reduction in space and eruption problems of succeeding teeth are possible effects on the dentition and thus may lead to an increase in orthodontic treatment need [8–15]. Malocclusion is a prevalent condition in Europe and other countries [18, 19]. The orthodontic effect of premature loss of deciduous teeth on malocclusion in the permanent dentition is not yet in the focus of studies [10, 17], even though it is an important aspect for ECC-related management and treatment decisions [10, 17].

The high prevalence and devastating consequences of ECC for the child, the family, its society and health care system indicate the need for early interventions in childhood [1–4]. ECC is an entirely preventable disease [1–4]. Although extensive research was performed on its management, the progress in the prevention has been slow [1–5, 20–27]. Dental caries is a non-communicable disease that shares common risk factors such as excessive sugar consumption [28]. For preventing ECC, a common vision of health and implementation of oral health strategies in child health programmes using new multi-sectoral and collaborative approaches is required [1, 3, 4, 28–30].

In this context, a regional programme for prevention (PP) of ECC in the German federal state of Thuringia was carried out. The programme starting at the time of birth focussed on a holistic approach for general and oral health promotion in young children and included primary, secondary and tertiary prevention measures. Basis was the cooperation between the Department of Preventive and Paediatric Dentistry, Jena University Hospital, and the communal newborn visiting service of the Youth Welfare Office of the city of Jena to gain access to all families. Caregivers were counselled about daily tooth brushing with fluoride toothpaste and reducing consumption of sugar and the early establishment of a medical and dental home using anticipatory guidance and motivational interviewing approaches [1–4, 20, 21, 31–35]. Additionally, the children were included in a caries-risk-related recall system with comprehensive continuous dental care [1–4, 36, 37]. Previous programme evaluation was carried out at the age of 1, 3 and 5 years [38–40].

The aim of this prospective birth cohort study was to evaluate the effect of the PP of ECC and the resulting need for orthodontic treatment in 8-year-old German children. The null hypothesis was that the PP was not effective for preventing caries and premature primary tooth loss in children. The study hypothesis was that children, who had been enrolled in a caries-risk-related recall system with continuous dental care starting at the time of birth, have a better oral health with less premature loss of deciduous teeth and lower need for orthodontic treatment at the age of 8 years than children who did not participate (the control group).

## Materials and methods

### Study design

This prospective birth cohort study was approved by the Ethics Committee of Jena University Hospital (2759-02/10; German Clinical Trials Register DRKS00003438). Conduction of the study was in full accordance with the ethical requirements of the World Medical Association Declaration of Helsinki (2008) and all caregivers had to give written informed consent to participate in the study. The study was complied with the recommendations of the STrengthening the Reporting of OBservational studies in Epidemiology (STROBE) statement guidelines.

### Preventive programme

The regional preventive programme (PP) was carried out in the German federal state of Thuringia and had been described previously [38–40]. The basis was the cooperation between the Department of Preventive and Paediatric Dentistry (DPD), Jena University Hospital, and the communal newborn visiting service (CNVS) of the Youth Welfare Office of the city of Jena to ensure access to all families for general and oral health promotion in young children. Since 2009, all parents of newborn children (about 1000 children per year) have been visited and counselled by CNVS-qualified staff (midwives, social workers, nurses) between the 1st and 4th weeks after birth. Mothers were counselled on general and oral health using brief motivational interviewing and anticipatory guidance approaches (covering the importance of breastfeeding, use of baby bottles and pacifiers, healthy diet, importance of screening examinations by a paediatrician and caries development and its prevention). Families were advised about the importance of daily tooth brushing with fluoride toothpaste, reducing consumption of sugar and the early establishment of a dental home. A dental home should be established in the first year of a child’s life followed by regular dental care. Every family received a folder with brief information material in their native language, and a toothbrush, fluoride toothpaste and a pacifier for the child were provided as incentives.

The children of the Jena birth cohort July 2009 to October 2010 (*n* = 1162) had the possibility to participate in the PP with caries-risk-related continuous dental care provided by a paediatric dentist in the dental practice of the DPD at least to the age of 5 years. Recruitment was done by the CNVS by sending invitations to all families for a dental examination in the first year of the child’s life. Five hundred and twelve families accepted the invitation and were included as participants of the PP (prevention group). Those families who did not appear for the dental examination were included in the control group (CG). It has to be mentioned that all families received the same maternal counselling at time after birth with the difference that families in the CG were personally responsible to establish a dental home with regular dental care.

The Caries-Risk Assessment Tool for infants, children and adolescents of the American Academy of Pediatric Dentistry (AAPD) was used to categorise the children and re-evaluated at each dental appointment [36]. Children with a low or moderate caries risk were reappointed every 6 months and children with an increased caries risk every 3 months [36, 37]. In children with a low or moderate caries risk, fluoride varnish (Fluoridin N5, VOCO GmbH, Cuxhaven, Germany; 0.25 ml) was applied biannually by a dentist in the dental practice from the age of 3 years [25, 27, 31–33, 41]. In the first 3 years of life, fluoride varnish application was limited to children with an increased caries risk. Those who are at low risk received a minimal amount of fluoride varnish application biannually, limited to surfaces at risk (< 0.25 ml), whereas in older high-risk children (> 3 years), fluoride varnish was applied quarterly [25, 27, 31–33, 41].

In 2013 and 2015, a programme evaluation was conducted and described [39, 40].

### Study population

**Fig. 1:**
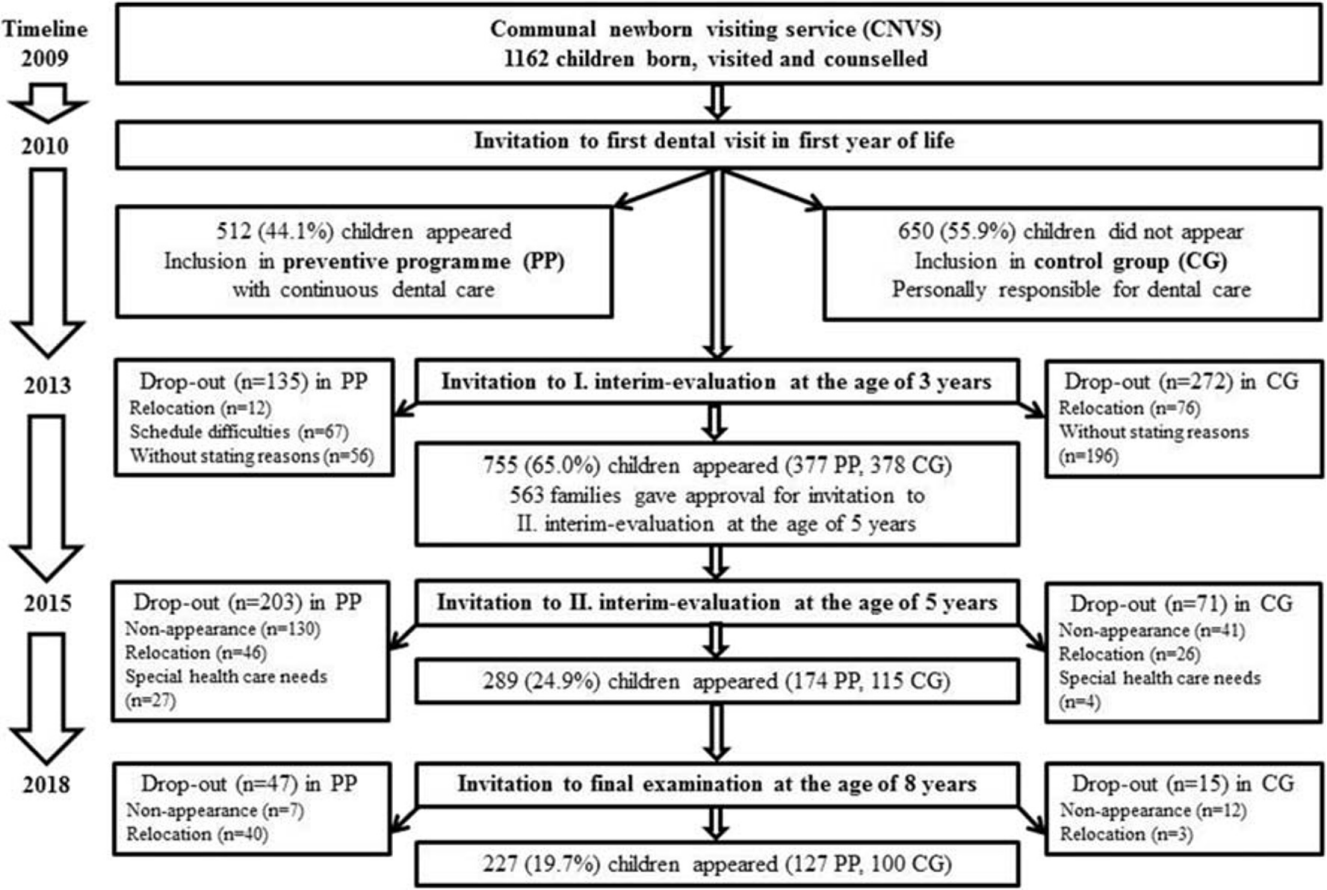
Participant flow diagram of the total birth cohort for the 8-year programme

In 2018, all families (*n* = 289) participating in the last programme evaluation at the age of 5 years [40] were invited to an examination in the DPD. Figure 1 presents the participant flow diagram of the total birth cohort for the 8-year PP. The study population included all children who followed the invitation. The eligibility criteria were the provision of written consent by the caregiver and the availability of data relating to the dental examination of the child. Children with special health care needs (congenital, developmental or acquired disability or impairment) and those with incomplete data and missing parent’s written consent were excluded from the analysis of the study.

Sample size calculation was done at the beginning of the prospective study. The sample size was calculated by dental caries experience based on the last nationwide survey of 6-year-olds in Germany in 2009, which was 2.56 dmft in Thuringia. To detect a 30% difference between the PG and CG with a two-tailed significance test, a 5% critical level and power of 80%, a sample of 66 children per group was required.

### Dental examination

Children were examined in the DPD by two blinded clinicians after professional tooth cleaning. All the examinations were conducted using a dental light, plane mouth mirror and a CPI ball-end probe. Caries experience was assessed by the dmfs index at the d1-level and scored according to the WHO standard criteria [42]. No radiographs were taken. In children with premature loss of one or more deciduous molars, conventional alginate impressions were taken for orthodontic study models of the dental arches and poured immediately in plaster. The bite was recorded using a wax wafer.

All records were performed by two trained and calibrated examiners. The calibration procedures were performed following the WHO guidelines with 10 children prior to the beginning of the survey [42]. Afterwards, the dentists examined a group of 20 pre-selected children twice on successive days to assess the consistency. The intrarater- and interrater-reliability was very good (*κ*_intra1_ = 0.89; *κ*_intra2_ = 0.92; *κ*_inter_ = 0.90).

### Orthodontic analysis

Orthodontic study models of children with premature loss of primary teeth were measured independently by two blinded, trained and calibrated orthodontists. Intra-arch measurements were made with a vernier calliper (Münchner Model 042-751-00, Dentaurum, Germany) with an accuracy of 0.1 mm. Study models were investigated to record the following parameters:

1. Arch lengths in millimetres in the incisal segment and lateral segments measured between the distal surfaces of the lateral incisors and from the distal surfaces of the lateral incisors to the mesial surfaces of the first molars. Extent of total space loss or space excess in accordance with Moyer’s mixed dentition space analysis [43] and Tonn’s tooth width analysis [44].
2. Orthodontic treatment need due to space loss based on the German KIG system (Kieferorthopädische Indikationsgruppen = Orthodontic Indication Groups) and the Index of Orthodontic Treatment Need (IOTN) [45]. The transfer of KIG classification to the IOTN was carried out using a specially designed scheme described by Streckbein [46]. A total space loss of over 3 and under 4 mm in the lateral segments was defined as P3 (KIG) or 5ipx (IOTN); a total space loss of at least 4 mm was defined as P4 (KIG) or 5ip (IOTN).

Both orthodontists measured each study model twice and means were calculated for each parameter. Prior to the beginning of the study, a calibration and training were conducted with the measurement of ten study models. Afterwards, both orthodontists independently measured 30 pre-selected study models to assess the consistency. The intraclass correlation coefficients for intraobserver agreement regarding measurement of space loss or space excess were 0.990–0.998 (IK) and 0.991–0.998 (TK). The intraclass correlation coefficients for interobserver reliability with regard to the space analysis were 0.954–0.996. The average measuring difference between the dentists was 0.05 mm.

### Additional information

Information regarding socioeconomic status (SES) of the families, group allocation (prevention or control group) and time of premature tooth loss were compared with the findings of the interim evaluation in 2013 and 2015 [39, 40, 47].

### Statistical analysis

Data were recorded in Microsoft Excel files (Office Version 2016, Microsoft Corporation, Redmond, WA, USA) and transferred to the Statistical Package for Social Sciences (SPSS version 23, IBM Corporation, Armonk, NY, USA) and GraphPad Prism (version 7, GraphPad software, San Diego, CA, USA) for analysis. The primary endpoint was the caries experience measured by the dmft/DMFT (decayed, missing and filled teeth) index. Secondary endpoints were the dmfs/DMFS index (including non-cavitated carious lesions), the caries prevalence, the prevalence of premature tooth loss and the extent of lack of space. Frequencies, percentages, mean values and standard deviations were calculated. The primary and secondary endpoints were compared between the two groups of children using chi-square tests, Fisher’s exact test or unpaired *t* test. The central limit theorem of statistics has been complied with. Group sizes below 30 children were graphically checked for normal distribution per group. The significance level for all statistical tests was set at *α* = 0.05. Additional the caries prevalence and caries experience related to a low socioeconomic status (SES) were calculated and compared between groups. The SES of the families was assessed by using the Brandenburg social index [47]. The index was computed for each child based on the education and employment status of their parents. The children were allocated to lower, middle or higher SES groups [47]. For cases with missing values for one parent, the value of the other parent part was double-weighted, analogous for single parents [47]. For data analysis and comparison of the prevention and control groups, a matching of all examined subjects relating to the variables (‘matching criteria’) of sex, age, SES and ethnicity was performed. In the case of several suitable statistical twins, the subjects were assigned at random to the control group. This matching reduced potential sources that might have influenced oral health outcomes.

## Results

The response rate was 78.5%. A total of 227 children (aged 8.4 ± 0.6 years; 45.8% female) were included in the study (PG *n* = 127, CG *n* = 100) with an average of 12.7 primary and 11.2 permanent teeth. Sixty-two children had to be excluded due to non-appearance on dental examination day (*n* = 19) and relocation to another area (*n* = 43). Figure 1 presents the participant flow diagram of the total birth cohort for the 8-year PP. Caries was present in 18.5% (*n* = 42) of the children. Prevalence of premature tooth loss was 22.5% (*n* = 51). There were no differences between genders (results not shown).

Description of all children according to group at baseline (time at birth) and at 8 years of age is presented in Table 1. Children in the PP had significantly lower caries prevalence and experience at the d_1–4_/D_1–4_- and d_3–4_/D_3–4_-level than children in the CG (*p* < 0.05; Table 2). The proportion of children with low SES was similar in both groups and the impact of SES on caries experience was significant (Table 2). Children with a low SES had the highest caries experience in the PG and the CG (PG 1.7 ± 1.5 d_3–4_mft; CG 8.8 ± 2.8 d_3–4_mft; Table 2). The data of caries prevalence, caries experience, prevalence of premature tooth loss, extent of space loss and orthodontic treatment need in the PG and the CG before and after matching are shown in Table 3. To avoid contamination biases, the total sample was parallelised. The final representative sample included 150 children after the matching process with 13.6% more than the estimated sample size.

**Table 1:** Description of the children according to group at baseline (time at birth) and at final examination

	Baseline				Final			
	Total	Prevention group (PG)	Control group (CG)	Fisher’s exact test *p* value	Total	Prevention group (PG)	Control group (CG)	Fisher’s exact test *p* value
Children (*n*)	1162	512	650		227	127	100	
Age in years	Time at birth				8.4 ± 0.6	8.4 ± 0.6	8.4 ± 0.6	1.000^#^
Male *N* (%)	595 (51.2)	266 (52.0)	329 (50.6)	0.679	123 (54.2)	63 (49.6)	60 (60.0)	0.140
Low SES *N* (%)	158 (13.6)	68 (13.2)	90 (13.8)	0.797	26 (11.5)	16 (12.6)	10 (10.0)	0.676
Caries prevalence (d3–4-level) *N* (%)	All children toothless				42 (18.5)	4 (3.1)	38 (37.3)	0.001*
d_3–4_mft (*x* ± SD)	All children toothless				2.0 ± 3.0	0.4 ± 1.0	3.9 ± 3.5	0.001^#^*
Caries prevalence (D3–4-level) *N* (%)	All children toothless				15 (6.6)	1 (0.8)	14 (14.0)	0.001*
D_3–4_MFT (*x* ± SD)	All children toothless				0.1 ± 0.5	0.0 ± 0.1	0.2 ± 0.7	0.002^#§^

^#^*t* test, *p < 0.001, ^§^*p* < 0.05

**Table 2:** Caries prevalence (%), caries experience (dmfs/dmft/DMFS/DMFT), prevalence of premature tooth loss, extent of space loss (mm) and orthodontic treatment need of the prevention and control groups and in children with low socioeconomic status (SES)

	Total	Prevention group (PG)	Control group (CG)	Low SES total	Low SES prevention group (PG)	Low SES control group (CG)
Children *N*	227	127	100	26	16	10
Caries prevalence (d1–4-level) *N* (%)	54 (23.8)	7 (5.5)°*	47 (47.0)	9 (34.6)	2 (12.5)°^§^	7 (70.0)
Caries prevalence (d3–4-level) *N* (%)	42 (18.5)	4 (3.1)°*	38 (38.0)	9 (34.6)	2 (12.5)°^§^	7 (70.0)
Caries prevalence (D1–4-level) *N* (%)	32 (14.1)	4 (3.1)°*	28 (28.0)	6 (23.1)	0 (0.0)°^§^	6 (60.0)
Caries prevalence (D3–4-level) *N* (%)	15 (6.6)	1 (0.8)°*	14 (14.0)	4 (15.4)	0 (0.0)°^§^	4 (40.0)
d_1–4_mfs (mean ± SD)	5.3 ± 8.6	1.1 ± 2.8^#^*	10.5 ± 10.3	13.1 ± 13.1	4.4 ± 4.4^#^*	27.0 ± 10.0
d_3–4_mfs (mean ± SD)	5.2 ± 8.5	1.1 ± 2.7^#^*	10.2 ± 10.2	13.0 ± 13.0	4.4 ± 4.4^#^*	26.7 ± 9.8
d_1–4_mft (mean ± SD)	2.2 ± 3.2	0.5 ± 1.0^#^*	4.2 ± 3.7	4.6 ± 4.3	1.8 ± 1.5^#^*	9.2 ± 3.1
d_3–4_mft (mean ± SD)	2.0 ± 3.0	0.4 ± 1.0^#^*	3.9 ± 3.5	4.4 ± 4.1	1.7 ± 1.5^#^*	8.8 ± 2.8
D_1–4_MFS (mean ± SD)	0.4 ± 1.2	0.1 ± 0.6^#^*	0.8 ± 1.7	0.6 ± 1.4	0.0 ± 0.0^#§^	1.6 ± 2.0
D_3–4_MFS (mean ± SD)	0.2 ± 0.8	0.1 ± 0.6^#^	0.3 ± 1.2	0.2 ± 0.6	0.0 ± 0.0^#§^	0.6 ± 0.8
D_1–4_MFT (mean ± SD)	0.3 ± 0.9	0.1 ± 0.4^#^*	0.6 ± 1.3	0.5 ± 1.2	0.0 ± 0.0^#§^	1.4 ± 1.6
D_3–4_MFT (mean ± SD)	0.1 ± 0.5	0.0 ± 0.1^#§^	0.2 ± 0.7	0.2 ± 0.5	0.0 ± 0.0^#§^	0.5 ± 0.7
Prevalence of premature tooth loss *N* (%)	51 (22.5)	10 (7.9)°*	41 (41.0)	13 (50)	3 (18.8)°^§^	10 (100)
Extent of space loss in mm	2.9 ± 4.2	0.4 ± 1.9^#§^	3.3 ± 4.4	5.4 ± 4.2	0.2 ± 2.5^#§^	6.4 ± 3.7
Orthodontic treatment need (%)	16 (7.0)	0 (0)°^§^	16 (16.0)	4 (15.4)	0 (0)°^§^	4 (40.0)

^#^*t* test, °Fisher’s exact test, *p < 0.001, ^§^*p* < 0.05

**Table 3:** Caries prevalence (%), caries experience (dmfs/DMFS), prevalence of premature tooth loss, extent of space loss (mm) and orthodontic treatment need (%) of the prevention and control group before and after matching

	Before matching					After matching				
	Total	Prevention group (PG)	Control group (CG)	Low SES prevention group (PG)	Low SES control group (CG)	Total	Prevention group (PP)	Control group (CG)	Low SES prevention group (PG)	Low SES control group (CG)
Children (*n*)	227	127	100	16	10	150	75	75	7	7
Caries prevalence (d1–4-level) *N* (%)	54 (23.8)	7 (5.5)°*	47 (47.0)	2 (12.5)°^§^	7 (70.0)	37 (24.7)	2 (2.7)	35 (46.7)	1 (14.3)	5 (71.4)
Caries prevalence (D1–4-level) *N* (%)	32 (14.1)	4 (3.1)°*	28 (28.0)	0 (0.0)°^§^	6 (60.0)	21 (14.0)	1 (1.3)	20 (26.7)	0 (0.0)°^§^	4 (57.1)
d_1–4_mfs (*x* ± SD)	5.3 ± 8.6	1.1 ± 2.8^#^*	10.5 ± 10.3	4.4 ± 4.4^#^*	27.0 ± 10.0	5.7 ± 9.0	0.6 ± 1.6	10.8 ± 10.5	3.6 ± 3.2	26.9 ± 10.6
d_1–4_mft (*x* ± SD)	2.2 ± 3.2	0.5 ± 1.0^#^*	4.2 ± 3.7	1.8 ± 1.5^#^*	9.2 ± 3.1	2.3 ± 3.4	0.3 ± 0.8	4.4 ± 3.8	1.6 ± 1.3	9.4 ± 3.6
D_1–4_MFS (*x* ± SD)	0.4 ± 0.9	0.1 ± 0.6^#^*	0.8 ± 1.7	0.0 ± 0.0^#§^	1.6 ± 2.0	0.0 ± 1.3	0.0 ± 0.1	0.8 ± 1.7	0.0 ± 0.0^#§^	0.9 ± 0.9
D_1–4_MFT (*x* ± SD)	0.3 ± 0.9	0.1 ± 0.4^#^*	0.6 ± 1.3	0.0 ± 0.0^#§^	1.4 ± 1.6	0.0 ± 0.9	0.0 ± 0.1	0.6 ± 1.2	0.0 ± 0.0^#§^	0.9 ± 0.9
Prevalence of premature tooth loss (%)	51 (22.5)	10 (7.9)°*	41 (41.0)	3 (18.8)°^§^	10 (100)	37 (24.7)	5 (6.7)	32 (42.7)	2 (28.6)	7 (100.0)
Extent of space loss in mm	2.9 ± 4.2	0.4 ± 1.9^#§^	3.3 ± 4.4	0.2 ± 2.5^#§^	6.4 ± 3.7	2.6 ± 3.7	1.0 ± 2.2^#^	2.8 ± 3.8	2.5 ± 0.3^#^	4.9 ± 3.1
Orthodontic treatment need (%)	16 (7.0)	0 (0)°^§^	16 (16.0)	0.0 (0)°^§^	4 (40.0)	6 (4.0)	0 (0)	6 (8.0)	0 (0)	3 (42.9)

^#^*t* test, °Fisher’s exact test, *p < 0.001, ^§^*p* < 0.05

### Orthodontic analysis

#### Early loss of deciduous teeth

**Fig. 2:**
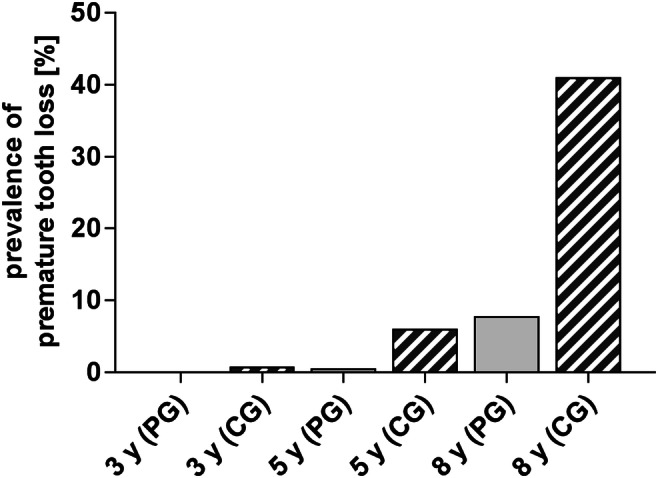
Prevalence of premature extraction of primary teeth of the total birth cohort for the 8-year programme. Prevalence in the prevention group (PG) and control group (CG) increased from the age of 3 years (y) to 5 years and to 8 years, but the amount was much higher in the CG (PG 0/0.6/7.9% vs. CG 0.8/6.1/41%)

Orthodontic analysis found a higher prevalence of premature tooth loss in the CG (41%) than in the PG (7.9%) group. The prevalence of tooth loss in both groups increased exponentially compared with previous examinations at the age of 3 and 5 years showing a significant higher amount of missing teeth in the CG group (Fig. 2).

**Fig. 3:**
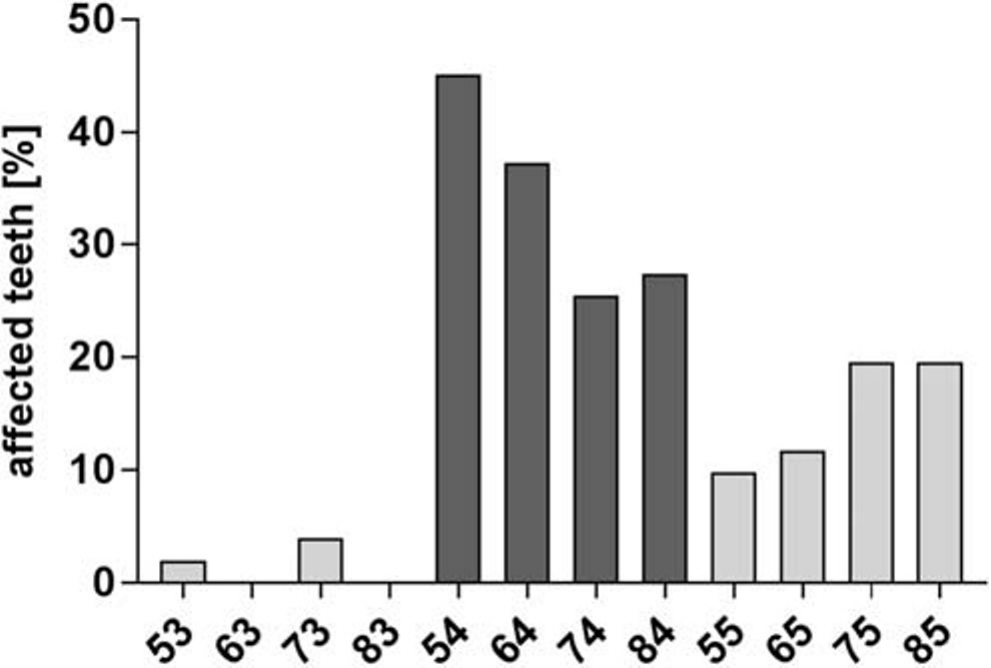
Distribution of premature extraction of primary teeth depending on the tooth type of the total birth cohort in 8- and 9-year-old children (*n* = 51); the most commonly missing teeth were the right upper primary first molars that were lost in almost every second child (45%)

The most common missing teeth were the right upper primary first molars (Fig. 3). Twenty children (39.2%) lost one primary tooth, 20 children (39.2%) lost two primary teeth, 4 children (7.8%) lost three primary teeth, 6 children (11.8%) lost four primary teeth and 1 child lost five primary teeth (2%). The prevalence for early loss of premature teeth was higher in the first primary molars compared with that in the second primary molars. Premature tooth loss was more frequently present in the upper (43.1%) compared with the lower jaw (29.4%). In most patients, only one jaw was affected. Premature tooth loss of both jaws was only present in the CG (27.5%) group, but not in the PG (0%) group.

#### Extent of tooth/arch length discrepancy

**Fig. 4:**
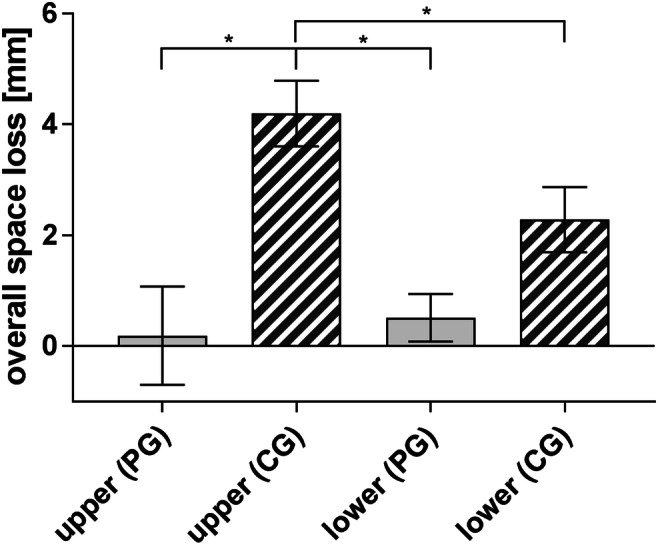
Extent of space loss in the prevention group (PG) and control group (CG) of the total birth cohort in 8- and 9-year-old children with premature extraction of primary teeth (*n* = 51) in the upper (PG 0.2 ± 3.3 vs. CG 4.2 ± 4.5 mm) and lower (PG 0.5 ± 1.5 vs. CG 2.3 ± 4.2 mm) jaws. Children in the CG showed a significant higher amount of space loss in the upper jaw than children in the PG. Statistical significant differences are marked with *p ≤ 0.05; means ± SEM

Space analysis revealed a significant higher extent of overall space loss in the CG (CG 3.3 ± 4.4 mm vs. PG 0.4 ± 1.9 mm) group (Fig. 4). Regarding the upper or lower jaw, space loss in the CG was significant higher in the upper (4.2 ± 4.5 mm) than in the lower jaw (2.3 ± 4.2 mm), whereas space loss in the PG was higher in the lower (0.5 ± 1.5 mm) than in the upper jaw (0.2 ± 3.3 mm), but the difference was not significant.

**Fig. 5:**
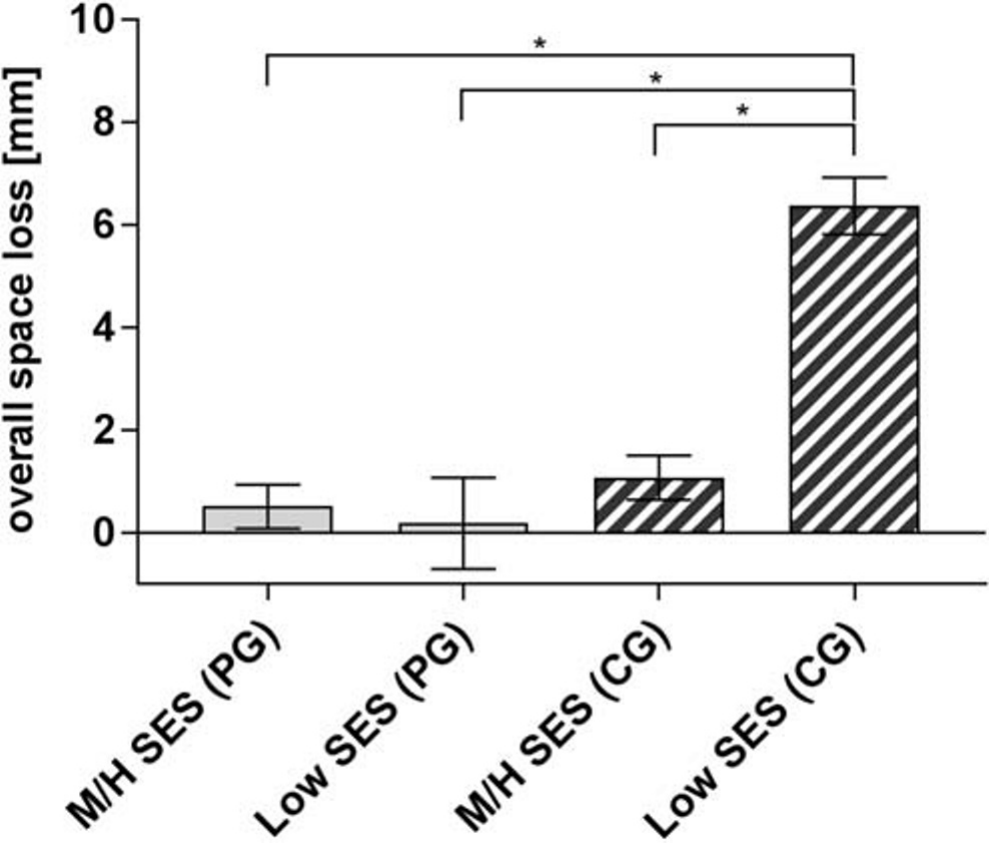
Extent of space loss in the prevention (PG) and control group (CG) of the total birth cohort in 8- and 9-year-old children with premature extraction of primary teeth (*n* = 51) regarding the socioeconomic status (SES). Children of the CG with a low SES showed a significant higher amount of space loss (6.4 ± 3.7 mm) than children of the PG (0.2 ± 2.5 mm) and children with a middle or high (M/H) SES in the PG (0.5 ± 1.3 mm) and CG (1.1 ± 3.4 mm). Statistical significant differences are marked with *p ≤ 0.05; means ± SEM

Children with a low SES showed a significant higher overall space loss (5.4 ± 4.2 mm) compared with children with a middle or high SES (1.0 ± 3.2 mm). Analysing the correlation of children’s SES and the effect of the dental care programme, the prevalence of observed *tooth*/*arch length discrepancies* demonstrated a significant increase if children who belonged to low SES and to the CG (Fig. 5).

#### Orthodontic treatment need

**Fig. 6:**
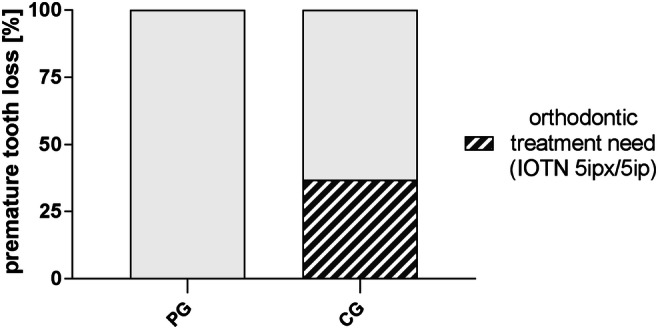
Orthodontic treatment need in children with premature extraction of primary teeth (*n* = 51) of the prevention (PG) and control group (CG) based on the IOTN 5ipx/5ip and KIG P3/P4 classification. Children who followed continuous dental observation showed a lower index for orthodontic treatment need because of significant space loss for lateral teeth (PG 0% vs. CG 36.8%)

Orthodontic treatment need due to the mesial drift of posterior teeth and space loss was present in 31.4% (*n* = 16) of the children with premature extraction of primary teeth (Table 2). Treatment need according to IOTN 5ipx/ip or KIG P3/4 index could be observed only in the CG group (Table 2; Fig. 6). Regarding the amount of space loss and arch length discrepancy, three children were categorised to P3 (KIG) or 5ipx (IOTN) and four children were categorised to P4 (KIG) or 5ip (IOTN). These findings are most likely associated with a severe extent of tooth/arch length discrepancy in the permanent dentition resulting in a high need for complex orthodontic treatment including interventions to create space in the lateral segment such as extractions of permanent premolars.

## Discussion

The evaluation of the long-term effect of the programme for prevention (PP) of early childhood caries (ECC) and the resulting need for orthodontic treatment in 8-year-old Thuringian children in Germany demonstrates that children who participate in the PP have a lower caries prevalence and caries experience than children who did not participate (the control group). The main results are that children who had been enrolled in a caries-risk-related recall system with continuous dental care starting at the time of birth have a better oral health with less premature extractions of deciduous teeth and lower need for early orthodontic treatment at the age of 8 years than children who did not participate (the control group).

### Study hypothesis

The present study is based on data from a regional birth cohort study in Germany. The results show that the programme is effective for the prevention of caries and premature tooth loss in children and the null hypothesis could be rejected. A programme starting at the time of birth and consisting of early maternal counselling, establishment of a dental home and inclusion of the children in a caries-risk-related recall system with continuous dental care and fluoride varnish application can prevent caries in the primary and in the permanent dentition.

### Oral health

The analysis reveals significant differences between the PP and CG if caries prevalence, caries experience, prevalence of premature tooth loss, extent of space loss and resulting orthodontic treatment need are considered.

The findings of the previous analyses demonstrated that families who participated in the PP had a better oral health and feeding behaviour [39, 40]. Families in the PP started earlier with tooth brushing, and their children received a regular second brush more often [39, 40]. Regular removal and control of the biofilm by daily brushing with fluoride toothpaste is essential for caries prevention and the best home-based measure [2–4, 22–24, 26, 27, 31–33]. In addition, children in the PP had an improved dietary behaviour with less in-between sugar-containing snacks and beverages per day and a shorter duration of the use of the bottle than others [39, 40]. The provision of preventive guidance within the first year of life by the communal newborn visiting service and following comprehensive continuous dental care were the key elements of the PP for ECC. The first published results showed that families in the PP visited the dentist in their first year of life, whereas children in the CG visited the dentist at a later age [39, 40]. The regular conducted caries-risk assessment allowed individual preventive measures including dietary and oral hygiene counselling and follow-up intervals. The findings clearly demonstrate that comprehensive continuous dental care with repeated maternal counselling about oral health promotion by use of anticipatory guidance and motivational interviewing approaches support patient self-management strategies and raise awareness of ECC [1–4, 20, 21, 34–37]. Primary preventive measures were complemented by professional plaque removal and fluoride varnish application to prevent caries development and its progression and to stimulate remineralisation of initial, non-cavitated carious lesions [25, 27, 31–33, 41, 48]. Fluoride varnish application is an appropriate measure to prevent and control caries and children in the PP received more fluoride varnish applications than children in the CG [25, 27, 31–33, 41, 48]. Cavitated carious lesions were treated conservatively and restored to prevent further progression and to avoid premature tooth loss. Previous analysis revealed that the care index in 5-year-old children in the CG was lower (45.1%) than that in the PP (100.0%) [39, 40]. Dental caries, if left untreated, can lead to further tooth breakdown, pulp exposure and premature tooth loss [1–4, 8].

### Premature tooth loss

The findings of the present and previous analyses show an increased prevalence of premature tooth loss with increased age in both CG and PP, but the amount was several times higher in the CG than in the PP [40]. The prevalence of 41.0% in the CG is comparable to previous literature with prevalence that varied between 22.3 and 65.4% depending on cohort and country [15, 49–51]. Participating in the PP led to a much lower prevalence of 7.9% and also to a delay of tooth loss of 2–3 years compared with the CG.

The PP had the additional benefit that in cases of premature tooth loss the space and dental development could be monitored and a space maintainer could be applied on demand. Studies show the importance of space maintainers because tooth migration begins within 3 weeks after tooth extraction and lasts for several months [52–54]. The present investigation shows that space loss was present if space management was left up to patients and parents and was not watched by professionals. Moreover, 31.4% space loss led to a subsequent need for orthodontic treatment defined by the KIG/IOTN classification.

### Preventive programme and oral health disparities

The PP approach and its regularity are responsible for the better oral health with a lower prevalence of premature tooth loss and less space loss in children in the PP compared with the CG. In addition, it led to a compensation of oral health disparities. Caries is particularly prevalent in socio-economically disadvantaged groups [55–57]. In this study, the socioeconomic status (SES) is also an influencing variable on caries development and resulting premature tooth loss. Even though the subgroups with a low SES are very small and the generalisability should be made carefully, it could be demonstrated that the PP has a positive effect on the dental health of 8-year-olds with a low SES. The SES-dependent disparities regarding caries experience, premature tooth loss and tooth/arch length discrepancies could be compensated. The same positive effect could be shown regarding caries prevalence and caries experience in the last programme evaluations [38–40].

### Strengths of the study

The PP of ECC already includes almost all recommendations of the current preventive and management strategies suggested by the IAPD Bangkok Declaration [1, 3]. The PP uses a new multi-sectoral approach providing preventive guidance within the first year of life by community health workers to raise awareness of ECC. The preventive measures included maternal counselling about reducing consumption of sugar, daily brushing with fluoridated toothpaste, the early establishment of a dental home, regular assessment of caries risk, inclusion of the children in a caries-risk-related recall system with comprehensive continuous dental care and fluoride varnish application. All children were drawn from the same population at time of birth, when no primary tooth has been erupted yet and followed over a period of 8 years. There were no baseline differences between groups regarding age, gender, SES and migration background [38–40]. For programme evaluation, children were surveyed at 3 and 5 years of age to capture oral health status [39, 40].

### Limitations

There are a few limitations of this study to note. First, this study was conducted in a medium-sized and well-situated city in Germany with a relatively low proportion of families with a low SES or migration background. Second, due to ethical considerations, all families should have the possibility to participate in the PP, so that the assignments to groups were made by the preference of the family and not at random. Consequently, the findings are restricted to this population group. Lower income groups usually have a lower response rate to health promotion and preventive programmes, which could represent a substantial risk of bias [55]. To reduce potential sources that might have influenced oral health outcomes, an analysis of the non-participants (*n* = 650) regarding their SES and ethnicity was carried out at the beginning of the study and showed no differences between the two groups (data not shown). Additionally, lack of randomisation was tried to compensate by an adequate statistical analysis including a representative sample size and a matching of all subjects. Another limitation is the participation rate in the examination. After 8 years, just 20% of the total birth cohort could be examined. Although the required number of children per group is representative and twice as high as in the sample size calculation, a generalisability of the study results has to be made with caution. The latter aspect is partly due to the fact that at the beginning 512 families decided to participate in the PP and 650 wanted to be personally responsible for establishing a dental home with regular dental care. The high drop-out rate can be explained by the long duration of the programme, relocation, schedule difficulties, effort and lack of interest. Nevertheless, 78.5% of the children of the last programme evaluation in 2015 could be examined. The last limitation is the precise determination of the time of premature tooth loss in children in the control group. While all data of the children in the prevention group were recorded longitudinally, starting at the time of birth to the age of 8, the data of the children in the control group could just be compared with the findings of the interim evaluation in 2013 and 2015 [39, 40]. To reduce the source of potential bias, the analysis relates to a defined period of time of premature tooth loss (before age 3, between age 3 and 5, between age 5 and 8).

### Summary

Children in the PP had a lower caries prevalence and caries experience, less premature loss of deciduous teeth and lower need for orthodontic treatment at the age of 8 years than children in the control group.

In summary, it can be stated that a programme consisting of the early establishment of a dental home, assessment of caries risk and inclusion of the children in a caries-risk-related recall system with continuous dental care and fluoride varnish application can prevent caries and premature tooth loss. In addition, children will show a reduced need for complex orthodontic interventions due to loss of space and the establishment of severe tooth/arch length discrepancies.
